# Illness beliefs among patients with chronic widespread pain - associations with self-reported health status, anxiety and depressive symptoms and impact of pain

**DOI:** 10.1186/s40359-017-0192-1

**Published:** 2017-07-05

**Authors:** P Järemo, M Arman, B Gerdle, B Larsson, K Gottberg

**Affiliations:** 1 Division of Nursing, Department of Neurobiology, Care Sciences and Society, Karolinska Institutet, S-141 83 Huddinge, Sweden; 2 Pain and Rehabilitation Centre, and Department of Medical and Health Sciences, Linköping University, Linköping, Sweden

**Keywords:** Illness beliefs, Chronic widespread pain, Self-rated health, Questionnaires, Anxiety and depression

## Abstract

**Background:**

Chronic widespread pain (CWP) is a disabling condition associated with a decrease in health. Illness beliefs are individual and are acquired during life. Constraining beliefs may prevent patients from regaining health. Understanding these patients’ illness beliefs may be a way to improve the health care they are offered. The aim of this study was to describe illness beliefs among patients with CWP and associations with self-reported health, anxiety and depressive symptoms, and impact of pain.

**Method:**

In this cross-sectional study, questionnaires were sent by mail to 330 patients including socio-demographic information, the Illness Perception Questionnaire (IPQ-R), the Short-Form General Health Survey (SF-36) and the Hospital Anxiety and Depression Scale (HADS). Data were analysed using descriptive statistics, non-parametric tests and linear regression analyses.

**Results:**

Patients experienced and related a high number of symptoms to CWP (mean (SD) 9 (3)). The patients believed their illness to be long lasting, to affect their emotional well being, and to have negative consequences for their lives. Some 72% reported having severe or very severe pain, and impact of pain according to SF-36 was negatively correlated to several illness beliefs dimensions, anxiety- and depressive symptoms. In regression analyses, the Identity, Consequences and Personal control dimensions of IPQ-R and Anxiety- and Depressive symptoms explained 32.6–56.1% of the variance in the two component scores of SF-36.

**Conclusion:**

Constraining illness beliefs in patients with CWP are related to worse health status, especially in cases of high number of physical or mental symptoms, beliefs of negative consequences or the illness affecting them emotionally. Identification and understanding of these beliefs may reduce patients’ suffering if they are taken into consideration in rehabilitation programs and in development of new evidence-based interventions aimed at increasing health in patients with CWP.

## Background

Chronic widespread pain (CWP) is a relatively common syndrome. Estimates for CWP prevalence were between 10 and 15% in the general population with twice as high prevalence in women than in men and higher prevalence among those aged over 40 [1, 2]. CWP is defined by The American College of Rheumatology [3] as pain in the axial skeleton, above and below the waist and on the left and right side of the body lasting more than 3 months. A more stringent definition, the “Manchester definition”, was developed by MacFarlane et al. [4] additionally requiring pain to be present in at least two of four sections of contralateral limbs.

“Illness beliefs” are individual and are acquired during life and during the course of an illness [5]. According to Wright, beliefs as a concept capture patients’ and health care providers’ efforts to make sense of an illness. Facilitating beliefs are beliefs that increase the possibilities of finding alternative solutions to manage an illness and hence soften illness suffering. Constraining beliefs are beliefs about the illness that can restrict options, maintain the problems and enhance illness suffering [5]. Beliefs have been implied to play an important role in living with illness since they can be determinants of patients’ health behaviour in managing illness [6, 7]. Previous research has shown that beliefs such as catastrophizing (constraining) and self-efficacy (facilitating) affect health in patients with CWP [8–10]. According to Wright et al. [5] other words such as ‘perception’, ‘cognitive representation’ and ‘explanation’ can be used synonymously with beliefs, but the term ‘belief’ is preferred as it best captures the individuals’ efforts to make sense of their illness.

Weinman et al. developed the Illness Perception Questionnaire – Revised (IPQ-R) [11, 12] in order to assess illness perceptions/beliefs based on a theoretical construction of perceptions/beliefs about illness and their impact on health behaviour [13]. As an example, Snelgrove et al. [14] described chronic pain patients viewing their pain as a biomechanical flaw, their physical body became the only focal point in managing the illness, which contributed to a limited effect of treatment.

Patients with fibromyalgia – a subcategory of CWP [3] – had difficulties in understanding their illness, had little personal control, low expectations of effective treatment and expected their illness to have a chronic course with serious consequences [10, 15]. Glattacker et al. [16] showed that beliefs of fewer consequences and fewer attributed symptoms led to better rehabilitation outcome. De Rooij et al. [17] saw improvement in negative beliefs to be a key agent of effect in multimodal treatment of patients with CWP.

Other factors which can influence and be influenced by illness beliefs are mood disorders and self-reported health status. Self-reported health status is the patients´ own perception and evaluation of health, a concept which is relevant in studying the consequences of disease and treatment [18]. Patients with CWP appear to have reduced self-reported health status [19] and studies of patients with chronic pain report a high prevalence of mood disorders ranging up to 80% [20, 21]. In order to reduce suffering, different alterable determinants of health status and mood disorders need to be identified and one of them may be the patients’ illness beliefs.

Thus, CWP is a disabling condition that impairs health status and is associated with a high economic and social burden for both the patients and the health care system, which illustrates the necessity of further knowledge. Swedish guidelines [22, 23] reflect the requirement for bio-psychosocial approaches to rehabilitation for musculoskeletal pain; therefore, understanding of patients’ illness beliefs and associated factors may be a way to improve care.

It was hypothesized that more constraining illness beliefs among patients with CWP are associated with decreased health status, but also taking anxiety- and depressive symptoms and impact of pain into account. Previous studies have not examined these dimensions simultaneously [8, 19–21, 24]. Hence, the aim of this study was to describe illness beliefs among patients with CWP. A further aim was to analyze associations between illness beliefs, anxiety- and depressive symptoms, impact of pain and mental- and physical health status.

## Methods

### Design

The design of the study was cross-sectional, and it included postal questionnaires. Power analysis gave an estimate of 128 respondents being sufficient for statistical analysis. Only reported data were included in analyses and missing data were not accounted for. Number of patients with CWP who reported data regarding all questionnaires is shown in Tables 1–5.

**Table 1:** Socio-demographic and clinical characteristics of patients with CWP (*n* = 152).

Gender n (%)	
Women	138 (91)
Men	14 (9)
Age (Yrs)	
Mean (SD)	46.3 (14)
Median (range)	46 (19–80)
Family situation n (%)	
Single	26 (17)
Single with children	14 (9)
Living with parents and siblings	4 (3)
Living with other adult with children	55 (36)
Living with other adult without children	53 (35)
Country of birth n (%)	
Sweden	134 (88)
European country excl. Sweden	13 (9)
Rest of the world	5 (3)
Education n(%)	
Low	24 (16)
Intermediate	91 (60)
High	23 (15)
Other	14 (9)
Work status n (%)	
Employed full time	28 (18)
Employed part time	9 (6)
Unemployed	22 (15)
Student	11 (17)
Sick leave	45 (30)
Disability pension	23 (15)
Retired	14 (9)
Occupational groups n (%)	
Administration and management	14 (9)
Health care, social care and commercial	52 (34)
Production and transport	19 (13)
Occupation requiring high or advanced education	16 (11)
Not specified	51 (34)
SF-36 Self-reported health, Mean (SD)	
BP bodily pain (n = 151)	24 (15)
PCS physical component score (n = 148)	28 (8)
MCS mental component score (n = 148)	36 (13)
Health transition (*n* = 151) n (%)	
Much better	5 (3)
Slightly better	12 (8)
The same	44 (29)
Slightly worse	49 (33)
Much worse	41 (27)
Form of pain (*n* = 143) n (%)	
Periodical	18 (13)
Persisting	125 (87)
Pain duration (*n* = 144) (Yrs)	
Mean (SD)	16 (11)
Median (range)	13 (2–49)
Pain according to Manchester definition n (%)	114 (75)
HADS Anxiety symptoms (*n* = 149) n (%)	
Cases	49 (33)
HADS Depressive symptoms (*n* = 148)	
Cases	48 (32)

### Participants

Patients were consecutively recruited from a pain and rehabilitation centre at a university hospital in the middle of Sweden during January 2011 to June 2013. Pain drawings and medical records were reviewed for inclusion criteria which were: to be at least 18 years of age, understand Swedish, and have CWP according to the Manchester definition [4] which requires pain to be present in the axial skeleton above and below the waist, in at least two sections of a limb in two contra lateral limbs. Medical records were reviewed for those patients whose pain drawing met the Manchester definition criteria. Eligible patients who met the basic inclusion criteria were 330 patients with CWP.

### Procedure

Data collection was made through patients answering questionnaires. An information letter was sent to the patients about the forthcoming study and 1 week later a letter indicating the purpose of the study, accompanied by the questionnaires sent to them by mail.

### Ethical considerations

The study was approved by the regional Ethics committee in Stockholm (2011/1384–31/3) and approval was obtained from the management of the pain and rehabilitation centre. The participants consented to participation in the study by returning the questionnaires.

### Socio-demographic- and pain characteristics

Information on age, gender, family situation, country of birth, education, work status, occupational group, spread of pain in the body, pain duration and form of pain (periodical/persisting) were self-reported background data. Spread of pain was indicated using a table with 18 boxes each for the left and right sides of the body. The patients marked the parts of the body where pain was present, to confirm the presence of pain according to the Manchester definition.

### Self-report measures

Illness beliefs were measured using the Swedish version of *Illness Perception Questionnaire - revised (IPQ-R)* [12, 25, 26]. The questionnaire includes an illness identity dimension, seven cognitive dimensions and a causal dimension. The first part measuring the illness Identity consists of a list of 14 symptoms. Patients rate whether they have experienced the symptom since their illness (yes/no) and whether the symptom is related to their illness (yes/no). The sum of the answers rated as ‘yes’ on the second question for each symptom forms the illness identity scale. A high sum indicates a stronger belief that the symptom is a part of the patients’ illness. The second part, exploring seven cognitive dimensions, consists of 38 items about beliefs concerning an acute/chronic *Timeline*, a cyclical *Timeline*, perceived Consequences of the illness and beliefs about *Personal control, Treatment control, Illness coherence* and *Emotional representations* (further described in Table 4). The third part, the Causal domain, consists of 18 items concerning causes of illness. This domain can be divided into four groups; psychological attributions (six items), risk attributions (seven items), immune attributions (three items) and chance attributions (two items) [12]. All 38 items of the IPQ-R are rated on a Likert scale ranging from 1 to 5. Additionally, at the end of the IPQ-R, patients are asked to write down in their own words the three most important causes of their illness, and are allowed to list causes not provided in the closed-ended list. The IPQ-R is a reliable and well-validated self-report questionnaire [12, 15, 27, 28].

The *Short-Form General Health Survey (SF-36; Swedish version)* was used to assess the patients self-reported health status [29–31]. The SF-36 is a questionnaire which includes 36 items covering eight domains: *physical functioning (PF), role of limitation due to physical health problems (RP), bodily pain (BP), general health (GH), vitality (VT), social functioning (SF), role of limitations due to emotional problems (RE) and mental health (MH)* which are summarized into separate physical component (PCS: PF, RP, BP, GH) and mental component (MCS: VT, SF, RE, MH) summary scores. A further single item concerns health transition over the past year. The physical component summary score measures patients’ abilities to perform simple everyday tasks and how much their pain and health in general interfere with their ability to work or perform other life roles. PCS also measures the extent of bodily pain experienced, a dimension consisting of two items, level of and impact of pain during the last 4 weeks (BP). The mental component measures the extent to which patients’ emotional state interferes with their ability to perform daily tasks and to socialize, and their level of psychological wellbeing. Higher scores on the SF-36 represent less affected health status. The Swedish version has been validated in a Swedish normative population [29]. The SF-36 data of the patients with CWP were compared with Swedish reference population data [32].

Anxiety and depressive symptoms was measured with the *Hospital Anxiety and Depression Scale (HADS)* [33], a questionnaire for assessing the presence and severity of anxiety and depressive symptoms in non-psychiatric settings. Two subscales, each containing seven items on a four-point Likert scale (ranging from 0 to 3) are summed separately to yield scores for anxiety and depression. The two subscales range from 0 to 21, higher scores indicating a greater likelihood of anxiety or depressive symptoms. A cut-off point of 11 was chosen for HADS to indicate a definite case. A study in a large Swedish population showed good psychometric properties [34].

### Data analysis

All data were analysed using SPSS 22.0. Descriptive statistics were used to present socio-demographic and clinical characteristics. For categorical variables, frequencies and percentages were calculated and means and SDs for continuous variables. In general data was summarized by using mean and SD and associations were examined with non-parametric tests. These methods were chosen since the data is on ordinal level and the present approach is also supported by comparing results from parametric tests and no important differences were detected. For comparisons of groups of patients with or without anxiety and depressive symptoms and the dimensions of beliefs measured by IPQ-R, a Mann Whitney U test was used for the ordinal data. For the purpose of examining correlations between dimensions of illness beliefs, anxiety and depressive symptoms, self-reported health status including PCS, MCS and bodily pain (BP), a Spearman correlation test was used [35]. To label the degree of the rank correlations, 0.2 was regarded as small, 0.5 as moderate and 0.8 as large [36]. The Summary Independent-Samples T Test was used for comparing SF-36 data with Swedish reference population. A probability value less than or equal to 0.05 was considered statistically significant. In the regression analyses, all significant correlations from the univariate correlation analyses were accounted for with all dimensions of illness beliefs, anxiety- and depressive symptoms as predictors. Two stepwise linear regression analyses were performed to predict PCS and MCS of SF-36 respectively.

## Results

### Socio-demographic- and clinical characteristics

Of 330 contacted patients, 152 responded (46%). The non-respondents had a mean age of 42.5 years (significantly younger than respondents, p 0.005) and 85% were women. Table 1 presents the socio- demographic and clinical data of the patients who had a mean age of 46.3 years (SD 13.6, range 19–80) and 91% were women. The two largest groups of patients were either on sick leave (30%) or working (24%), whereof 6% were working part time. The vast majority of the patients had either an occupation in service, care and commercial work (34%) or had not specified their occupation (34%) [37]. The vast majority of the patients had education from upper secondary level (59%). Most of the patients were of Swedish origin (88%). Housing was shared for 83% of the patients and 17% lived alone. Pain was persistent for 87% and periodical for 13% of the patients. The median for pain duration was 13 years (range 2–49). At the time of answering the questionnaire 75% of the patients reported pain according to the Manchester definition.

### Illness beliefs

In the first domain, the illness *Identity* domain, patients reported experiencing a mean of nine different symptoms (SD 3.0). Of the symptoms experienced, a mean of eight symptoms (SD 3.3) were perceived to be related to CWP. Of the symptoms experienced, pain, fatigue, loss of strength, stiff joints and sleep difficulties were the most common and these symptoms were frequently related to CWP. Nine of the 145 patients (6%) who experienced pain and 12 of the 139 (9%) who experienced fatigue did not relate these symptoms to CWP (Table 2).

**Table 2:** Illness Identity dimension of IPQ-R: 14 commonly experienced symptoms in patients with CWP (*n* = 152)

Symptoms	Experienced, N (%)^a^	Related to CWP, N (%)^a^
Pain	145 (100)	136/145 (94)
Fatigue	139 (97)	127/139 (91)
Loss of strength	136 (98)	131/136 (96)
Stiff joints	131 (92)	121/131 (92)
Sleep difficulties	126 (91)	118/126 (94)
Dizziness	98 (73)	82/98 (84)
Headaches	97 (71)	85/97 (88)
Upset stomach	89 (65)	71/89 (80)
Breathlessness	70 (52)	52/70 (74)
Nausea	65 (50)	48/65 (74)
Wheeziness	63 (47)	43/63 (68)
Sore throat	34 (27)	9/34 (26)
Weight loss	25 (19)	18/25 (72)
Sore eyes	23 (15)	18/23 (78)

^a^% of those who experienced the symptom

The *Identity* dimension and the second domain with the seven cognitive dimensions of IPQ-R are shown in Table 3. The three highest scored dimensions were *Timeline acute/chronic, Consequences and Emotional representations.*

**Table 3:** IPQ-R dimensions in patients with CWP (*n* = 152)

	Mean (SD), range	Possible range
Identity, *n* = 95	8.0 (2.5), 0–14	0–14
Timeline acute/chronic, *n* = 143	26.6 (3.7), 14–30	6–30
Timeline cyclic, *n* = 149	14.0 (3.7), 4–20	4–20
Consequences, *n* = 145	21.6 (4.4), 8–30	6–30
Personal control, *n* = 141	17.7 (4.0), 8–30	6–30
Treatment control, *n* = 145	14.1 (3.4), 5–25	5–25
Illness coherence, *n* = 144	17.6 (5.3), 5–25	5–25
Emotional representation, *n* = 145	18.7 (5.3), 6–30	6–30
Subscale	High score for the dimension indicates a belief…	
Identity	that the symptoms are part of the illness	
Timeline acute/chronic	that the illness is permanent rather than temporary	
Timeline cyclical	that the illness is cyclical in nature	
Consequences	that the illness has negative consequences	
Personal control	of good personal control over symptoms	
Treatment control	that the illness is amenable to treatment	
Illness coherence	of personal understanding of the illness	
Emotional representation	that the illness will affect the emotional well being	

In the third domain, the *Causal* domain, psychological factors (e.g. stress, worry, overwork, emotional state) and risk factors (e.g. heredity, poor medical care, own behaviour) were the attributions with which most of the patients agreed/strongly agreed. In the part where patients could write down their own beliefs about the most important causes of their illness they indicated mainly psychological factors such as stressful events in life, work-related stress and risk factors such as heredity and accidents related to work and traffic.

### Health status, anxiety and depressive symptoms and impact of pain

Health status, anxiety and depressive symptoms and impact of pain in patients with CWP are presented in Table 1. The PCS score in SF-36 had a mean (SD) of 28 (8) and the MCS score mean (SD) was 36 (13). In the item concerning health transition during the past year (Item 2, SF-36), 11% reported that their health was better, 29% reported their health was the same, 33% that their health was slightly worse and 27% that their health was much worse. The health status in patients with CWP was significantly (*p* < 0.001) worse than in a reference population in all dimensions [32]. About a third of the patients rated themselves as having anxiety (33%) and depressive (32%) symptoms according to the two subscales of HADS (Table 1). Regarding the impact of pain, (from item 7 and 8 in SF-36), where the patients could indicate how much pain they had experienced during the last 4 weeks, 72% reported having severe or very severe pain, and 27% reported that the pain interfered extremely with their normal work (Table 1).

### Associations between patients’ illness beliefs, health-status, anxiety and depressive symptoms, and impact of pain

We found several significant small to moderate correlations among the dimensions studied (Table 4). Patients who reported more symptoms related to their illness (*Identity*) and believed their illness to have negative *Consequences* rated PCS and MCS low and reported more impact of pain. The more the patients believed having *Personal control* over their illness and that their illness was amenable to treatment (*Treatment control*), the higher they rated PCS and the lower the impact of pain reported. The more the patients believed the illness would affect their emotional well-being (*Emotional representation*) and the more anxiety and depressive symptoms they experienced, the lower they rated MCS and the higher the impact of pain reported (Table 4).

**Table 4:** Correlations^a^ between dimensions of IPQ-R^b^, HADS^c^ and SF-36^d^ in patients with CWP (*n* = 152)

IPQ-R dimensions		PCS	MCS	BP^e^
Identity	*n* = 95	−.278*	−.370**	−.410**
Timeline acute/chronic	*n* = 143	−.126	−.120	−.130
Timeline cyclic	*n* = 149	.108	−.052	.064
Consequences	*n* = 145	−.360**	−.484**	−.473**
Personal control	*n* = 141	.299**	.129	.234**
Treatment control	*n* = 145	.216*	.097	.165*
Illness coherence	*n* = 144	.072	.044	.108
Emotional representation	*n* = 145	−.036	−.570**	−.244**
Anxiety symptoms	*n* = 149	.126	−.729**	−.231**
Depressive symptoms	*n* = 148	−.047	−.788**	−.374**

^a^Spearman’s Rank Order Correlation

^b^IPQ-R, Illness Perception Questionnaire-Revised

^c^HADS, Hospital Anxiety and Depression Scale

^d^SF-36, Short-Form General Health Survey; MCS, Mental Component Summary  score; PCS, Physical Component Summary  score; BP, Bodily Pain

^e^Bodily Pain is a dimension of PCS

*p < 0.05

**p < 0.01

Relating a high number of symptoms to their illness (*Identity*) was significantly associated with the presence of anxiety (*p* < 0.001) and depressive symptoms (*p* < 0.045). Furthermore, believing that the illness would have negative *Consequences* on their lives and that it would affect them emotionally (*Emotional representation*) were significantly associated with the presence of anxiety and depression (*p* < 0.001).

In regression analyses the *Identity, Consequences and Personal control* dimensions explained 32.6% of the variance in PCS (F = 4.368; *p* = 0.040). Thus *Consequences* dimension and Anxiety and Depressive symptoms explained 56.1% of the variance in MCS (F = 5.248; *p* = 0.025) (Table 5).

**Table 5:** Summary of stepwise multiple regression analyses for the prediction of SF-36 (PCS and MCS) by the illness beliefs dimensions^a^ and anxiety and depressive symptoms^b^

Significant predictors	B	CI 95%	p
Physical Health*			
Constant	33.706	22.197–45.215	0.000
Identity	−0.833	−1.627 - -0.040	0.040
Personal control	0.663	0.286–1.040	0.001
Consequences	−0.487	−0.924 - -0.051	0.029
Mental Health**			
Constant	55.867	45.223–66.512	0.000
Consequences	−0.619	−1.157 - -0.081	0.025
Depressive symptoms	−11.143	−16.280 - -6.005	0.000
Anxiety symptoms	−10.366	−15.232 - -5.501	0.000

*ΔR^2^ = 0.326 **ΔR^2^ = 0.561

F = 4.368 F = 5.248

*p* = 0.040* *p* = 0.025*

*n* = 78 *n* = 81

^a^IPQ-R

^b^HADS

## Discussions and conclusions

The aim of the present study was to describe illness beliefs among patients with CWP and their association with self-reported health, anxiety and depressive symptoms and impact of pain. The majority of patients in this study were women (91%), which is consistent with characteristics of populations with CWP [1, 38]. At inclusion, all patients reported pain through pain drawings in their medical records according to the Manchester definition, but during analysis of self-reported pain in the protocol 25% no longer fulfilled this definition and 13% indicated having periodic pain. This may be due to the fluctuation of symptoms, indicating that CWP is not necessarily a constant state [39].

On a public social level, pain influences productivity, as shown in a Swedish population study where chronic pain in age groups below the age of 65 was strongly associated with a lower prevalence of working [1]. In the present study, 24% of the patients with CWP were working compared with 57% in a study of patients with fibromyalgia [10]. This might have been because the patients in the present study had longer illness duration.

In the *Identity* domain of IPQ-R, all symptoms were endorsed by at least 15% of the patients, confirming the validity of the symptoms included in the domain. A mean of eight out of 14 symptoms was endorsed by the patients as being related to their illness. Pain, fatigue, loss of strength, stiff joints and sleep difficulties were related to CWP by over 90% of the patients, which is consistent with other studies [10, 15, 16]. Nine of the patients who reported having pain did not relate it to their illness, perhaps experiencing pain of another origin than CWP. Furthermore, patients who had recently become ill might not yet relate pain symptoms to the diagnosis of CWP.

The *Timeline acute/chronic*, the *Consequences* and *Emotional representations* were the three highest rated dimensions of illness beliefs. These findings are not surprising since CWP is a chronic condition, which has reached a permanent level for those who have had the illness a long time i.e. a reported mean time of 16 years. Furthermore, during the course of having CWP for a long period of time, the patients may have experienced disabling consequences and learned that the illness affects their emotional well being. The description of high scores in the original IPQ-R version does not provide a cut-off point. In comparison with other patient groups [25, 40, 41] CWP patients had stronger beliefs of their illness to be chronic and permanent and their pain as having more serious *Consequences* on their life. Furthermore they had less sense of *Personal- and Treatment control*. On the *Emotional representation* scale, patients with CWP reported similar emotional impact of their illness as patients with cancer. Further studies could explore why CWP patients experience low *Personal- and Treatment control*, and the fact that they believe to the same extent as patients with potentially mortal conditions that their illness will have negative *Consequences* for their well-being. The severity of the illness CWP, from the perspective of patients, is notable.

Psychological causes, including experiences of stress and work-related stress, were the most reported *Causes* of the patients’ CWP. There are conflicting results in studies showing psychosocial aspects in addition to chance and biological causes as the most prominent causes [10, 15, 42]. The psychosocial aspects might reflect patterns in society where people have been found to be unable to handle difficulties in an increasingly complex and stressful life and where life problems are somaticized and medicalized [43]. Thus, interventions supporting patients managing the complexity of living with CWP could be essential.

In univariate and multivariate association analyses, the dimensions of *Consequences, Identity, Personal control*, and Anxiety- and Depressive symptoms predicted health-status significantly and independently of each other. In addition *Consequences* was the dimension that independently was associated with both PCS and MCS. In concordance with other studies [10], patients had strong beliefs about the illness having negative Consequences, and the stronger the beliefs in this area were, the lower the degree of physical and mental health (PCS and MCS). The more the patients believed the illness would have negative Consequences and affect their mental wellbeing (Emotional representation) the more they expressed having anxiety and depressive symptoms. There are difficulties assessing whether anxiety and depressive symptoms are pre-existing, favouring the development of chronic pain, or a consequence of the chronic pain [44]. The stronger the belief in *Personal control* over symptoms and that illness is amenable to treatment (*Treatment control*), the higher patients rated their physical health and the less impact of pain they reported. de Rooij et al. [17] found that strong beliefs about *Personal* and *Treatment control* were associated with improved outcome of rehabilitation. These beliefs could be strengthened through care and bio-psychosocial rehabilitation with more patient involvement and through sharing views on how to manage the condition [17]. The more symptoms (*Identity*) patients related to their illness, the more they experienced anxiety and depressive symptoms, the lower they rated their physical and mental health, and the greater the impact of pain they reported, all of which is consistent with previous research (14, 22). Some 30% of the patients had considerable anxiety and depressive symptoms, which is in line with earlier studies demonstrating substantial rates of mood disorders in chronic pain patients [20, 21, 45, 46]. Ovemeer et al. [47] found in a study of patients with back-pain, that distress and negative emotions probably prevented them from benefiting from the offered bio-psychosocial treatment. When their constraining beliefs were not challenged but persisted they were consequently at risk of higher disability. This might be one explanation for why such a large proportion as 60% in the present study reported their health as worse than a year ago (data from the SF-36) even though they had had contact with the pain clinic. As hypothesized, constraining beliefs in patients with CWP were associated with decreased health status with anxiety- and depressive symptoms accounted for.

Generic rather than illness-specific instruments for examining illness beliefs and self-reported health were used, which was nevertheless well suited to this group of respondents since chronic pain patients were included in the development of IPQ-R [12] and SF-36 is a generic instrument considered to be useful in most patient groups [29]. IPQ-R was chosen for examining beliefs because it was easy to access, had been translated into Swedish, had a sound theoretical background, and is one of the most validated and most frequently used measures for examining illness beliefs. In the present study, all parts of IPQ-R were used to give a fuller description of the beliefs that patients with CWP held. However, some limitations should be considered, such as the large number of items, which might be difficult to complete, thereby entailing a risk of missing items. Self-reporting by mail is convenient but limited by the respondents’ ability to comprehend and is a risk for a higher non-response bias, although the method provides ample time for completing the questionnaire. In the present study the response rate was 46%, which might limit the generalizability.

The items in the *Identity* domain seems to be difficult to complete which may be because the option of “I do not know” is missing, which might explain some of the missing data. The non-respondents were significantly younger than the respondents. Young patients and other patients who have not experienced the severity of their illness long enough might not identify themselves as CWP patients, which may have affected participation in the study. Furthermore, the present patient group is not matched in the comparison of results with other illness groups and the results of the comparison should be interpreted only as an estimate. Comorbidities might influence illness beliefs but were not assessed in this study. Additional factors that may have affected the results are that, before answering the questionnaire, some patients may have received care or treatment in connection with the visit to the pain clinic, such as bio-psychosocial rehabilitation or events in daily life that were not controlled for in the present study. Missing data were not accounted for and in the regression analysis the number of patients decreased when the dimension of *Identity* was included, which may have affected the result. The design of this study does not allow conclusions to be drawn regarding causal relationships.

Illness beliefs have been shown to predict the outcome of treatment [48], to change over time [49] and they may be improved by treatment [50]. Furthermore, the beliefs of an illness rather than the symptoms themselves have been shown to account for patients´ illness adaptation [51]. The results of this study show that besides examining pain characteristics and the impact of pain, illness beliefs of patients and level of anxiety and depression are important to address when offering rehabilitation for patients with CWP. Illness beliefs may be regarded as a personal factor [22, 52] that influences health and interacts with functioning, and may facilitate understanding differences in how patients are managing their illness. Thus, strengthening facilitating illness beliefs and challenging constraining beliefs could be of vital importance in the rehabilitation of patients with CWP, helping them to maintain and improve their health. The effect of such rehabilitation taking illness beliefs into account should therefore be scientifically evaluated. Future studies could further examine the relationships between the factors studied in the present research, including the impact of illness beliefs on health status in the longitudinal perspective.

The findings of this study show that the patients had several constraining beliefs about their CWP that were related to worse health-status, especially in cases of high numbers of attributed physical or mental symptoms. Illness beliefs are important to determine because patients act according to their beliefs [5, 6]. Therefore, according to the findings in this study, patients need support in understanding CWP and in managing the psychological factors and risk factors they believed caused their CWP. The patients additionally need emotional support and involvement in treatment to manage various symptoms and types of pain. Control over symptoms and reduction of negative consequences of living with CWP are essential since these beliefs were shown to affect both mental and physical health and to increase the impact of pain. Finding out the patients’ illness beliefs may facilitate understanding of their previous attempts to manage their illness and customization of individual treatment in rehabilitation programs, and may help in the development of new evidence-based interventions.
